# Psychosocial competencies and reasons for living in young adults: implications for suicide prevention from a social determinants of health perspective

**DOI:** 10.3389/fpubh.2026.1810162

**Published:** 2026-04-14

**Authors:** Agnieszka Nieradko-Heluszko, Aleksandra Rył, Marta Giezek, Beata Karakiewicz

**Affiliations:** 1 Subdepartment of Social Medicine and Public Health, Department of Social Medicine, Pomeranian Medical University, Szczecin, Poland; 2 Department of Medical Rehabilitation and Clinical Physiotherapy, Pomeranian Medical University, Szczecin, Poland

**Keywords:** mental health, psychosocial competencies, reasons for living, social determinants of health, suicide prevention, young adults

## Abstract

**Introduction:**

Suicide prevention research has predominantly focused on risk factors, while psychosocial resources that may serve as protective factors have received less attention. From the perspective of social determinants of health, examining such resources may contribute to a more comprehensive understanding of mental health. This study aimed to analyze the relationships between psychosocial competencies and reasons for living among young adults, taking into account selected socio-demographic and family factors.

**Methods:**

A cross-sectional study was conducted between March and June 2025 among 546 young adults aged 18–35 in Poland. Reasons for living were assessed using the Reasons for Living Scale (RFL-48), including six subscales. Psychosocial competencies were evaluated across four domains: interpersonal skills, self-awareness, decision-making, and coping with stress. Hierarchical multiple regression analyses were performed, with socio-demographic and family variables entered in the first block and psychosocial competencies in the second.

**Results:**

Psychosocial competencies were significantly associated with reasons for living after controlling for socio-demographic and family factors. Self-awareness showed the strongest association with the overall reasons for living score and with survival and coping beliefs. Coping with stress was associated with survival and coping beliefs and with fear of suicide. Interpersonal skills and self-awareness were associated with fear of social disapproval, while self-awareness and decision-making were related to responsibility toward family.

**Conclusion:**

Psychosocial competencies are associated with various dimensions of reasons for living among young adults, partly independently of socio-demographic and family factors. These findings indicate that psychosocial competencies may be considered as relevant resources in the context of mental health and suicide prevention.

## Introduction

Suicidal behavior remains one of the most serious public health challenges worldwide, accounting for nearly 800,000 deaths each year and many more suicide attempts (1). In Europe and in many middle- and high-income countries, suicide is among the leading causes of premature mortality in young adults, generating significant social, economic and health consequences. In the European Union, suicide and intentional self-harm accounted for a significant proportion of deaths in the 15–29 age group, highlighting the scale of the problem in Europe (2). Global disease burden analyses confirm that suicide remains one of the leading causes of death among young people, and its consequences extend far beyond the individual level, affecting families, local communities and health systems (3). In Poland, despite a decline in the number of suicides in recent years, the incidence of suicide attempts remains high, indicating the persistence of the problem and the insufficient effectiveness of existing prevention strategies (4, 5). These data confirm the validity of in-depth analyses of protective mechanisms, especially in the young adult population, which is in a period of particular developmental sensitivity. Previous research and preventive measures in the field of suicidology have been largely dominated by an approach focused on risk factors such as depression, mental disorders, experiences of violence, addictions and relationship crises (6, 7). Although this perspective has provided important diagnostic data, it is increasingly argued that focusing solely on risk leads to an incomplete picture of mental health and limits the possibilities for designing effective interventions at the social and population level (8–10). According to the social determinants of health paradigm, mental health is the result of a dynamic interaction between material conditions, social relationships, cultural norms, and individual resources that enable effective coping with the challenges of everyday life (11, 12). Within the WHO social determinants of health framework, structural determinants refer to broader socio-economic and political conditions that shape social position, such as education, employment, income, and social inequalities, whereas intermediate determinants include living conditions, social support, family environment, and psychosocial circumstances directly influencing everyday functioning and health outcomes (13). In this perspective, psychosocial competencies may be understood as individual resources operating at the intermediate level, because they shape how individuals respond to social stressors and use available social and environmental resources in everyday life. In this approach, increasing attention is being paid to protective factors and psychosocial resources that can mitigate the impact of stressors and reduce vulnerability to suicidal behavior. The literature is paying increasing attention to the population of young adults who are undergoing intense educational, professional and family changes. This period is characterized by a simultaneous increase in autonomy and exposure to economic instability, social pressure and uncertainty about the future, which is conducive to mental crises (14, 15). At the same time, young adulthood is a crucial period for the formation of lasting psychosocial resources that may serve as protective factors in later life (16). This developmental stage is also marked by identity consolidation, increasing responsibility for independent life decisions, and reduced external support, which together may intensify vulnerability to psychological crises while simultaneously shaping long-term coping patterns. At this stage, reasons for living may be particularly relevant because personal values, future goals, interpersonal commitments, and emerging social roles are still being actively consolidated, making protective cognitions especially important in situations of psychological distress. Within suicidology, reasons for living are understood as cognitive and emotional protective resources that may buffer suicidal ideation by strengthening beliefs, values, and commitments supporting continued life engagement. In Linehan's framework, reasons for living constitute protective cognitions that counterbalance suicidal thoughts and reduce the likelihood that suicidal crises translate into action (17). The structure of reasons for living in this age group is strongly conditioned by culture and society, which emphasizes the need for analyses conducted in different national contexts (18). In parallel with research on reasons for living, social science literature is paying increasing attention to psychosocial competencies, also referred to as life skills. Psychosocial competencies are understood in this study as a set of cognitive, emotional, and interpersonal abilities that support adaptive functioning in everyday life and enable effective coping with developmental and social challenges. This understanding follows the WHO life skills framework, in which psychosocial competencies include skills such as self-awareness, interpersonal communication, decision-making, and coping with stress. They may also be interpreted as modifiable personal resources that support mental health and resilience. In the present study, psychosocial competencies are operationalized through four domains: interpersonal skills, self-awareness, decision-making, and coping with stress (19). These competencies are recognized as resources that can be developed through educational and environmental interventions and as important elements in the prevention of mental health problems (20, 39–41). From the perspective of the Interpersonal Theory of Suicide, psychosocial competencies may also be interpreted as resources potentially reducing perceived burdensomeness and thwarted belongingness, two interpersonal states regarded as central mechanisms contributing to suicidal ideation (21). Despite the growing importance of both areas, previous studies have usually examined psychosocial competencies and reasons for living as separate protective constructs, with limited evidence on how specific domains of psychosocial competence are associated with particular dimensions of reasons for living in young adults while accounting for socio-demographic conditions (22). The aim of this study was to analyse associations between psychosocial competencies and reasons for living in young adults, taking into account selected socio-demographic factors within the social determinants of health framework. By using multivariate analytical models, this study contributes to the literature by providing empirical evidence useful in the context of designing preventive interventions aimed at strengthening psychosocial resources related to reasons for living. The hypotheses were derived from the assumption that regulatory competencies such as decision-making and coping with stress are more directly related to internal protective beliefs, whereas interpersonal skills and self-awareness may be more closely linked to socially embedded dimensions of reasons for living, such as family responsibility and sensitivity to social evaluation. This assumption was based on the premise that decision-making and coping with stress directly support appraisal of difficult situations, perceived controllability, and the selection of adaptive responses, which are central mechanisms underlying survival and coping beliefs. Interpersonal competence and self-awareness were also considered relevant to family responsibility and sensitivity to social evaluation because they facilitate awareness of relational consequences, responsiveness to interpersonal expectations, and regulation of behavior in socially significant contexts. Based on a review of the literature, the following research hypotheses were formulated:

H1. A higher level of competence in decision-making and coping with stress will be associated with a higher overall level of reasons for living and with stronger Survival and Coping Beliefs (SCB).

H2. A higher level of interpersonal competence and self-awareness will be associated with a stronger sense of responsibility toward the family and greater sensitivity to social evaluation.

H3. The relationships between psychosocial competencies and reasons for living will remain evident even after taking into account selected socio-demographic conditions.

## Methods

### Study design and participants

The study had a cross-sectional design and used a questionnaire survey. The sample included *N* = 546 young adults residing in Poland. The study included 477 women (87.36%) and 69 men (12.64%). The marked predominance of women in the sample most likely reflects the characteristics of voluntary online recruitment, as differential response patterns and non-response bias in survey participation are well documented in methodology research (23). The study included individuals aged 18–35 years. Age was recorded categorically during questionnaire construction because it served primarily to verify inclusion within the predefined developmental group, which did not allow calculation of mean age and standard deviation. Most respondents lived in cities (72.53%, *n* = 396), while the rest lived in rural areas (27.47%, *n* = 150). The most frequently declared form of residence was the family home (71.25%, *n* = 389), followed by a rented flat (25.82%, *n* = 141), a student dormitory (2.20%, *n* = 12) and a room provided by a family member (0.73%, *n* = 4). In terms of family structure, the majority of participants were raised by both parents (80.95%, *n* = 442), 16.12% (*n* = 88) by their mother, and the rest by their father, grandparents or in foster care. The largest group in terms of education were respondents with secondary education 48.53% (*n* = 265), followed by those with a bachelor's degree 26.92% **(*n*** **=** **147)** and a master's degree 21.98% (*n* = 120). At the time of the study, 39.19% (*n* = 214) were married, 31.50% (*n* = 172) were in a civil partnership, and 29.30% (*n* = 160) were single. Participants were informed about the purpose of the study, the rules of anonymity and how the data would be used. Additionally, Table 1 presents the means (*M*) and standard deviations (*SD*) for assessments of financial situation, housing conditions, and frequency of domestic violence.

**Table 1:** Socio-demographic characteristics of the study sample (*N* = 546).

Variable	*n*	%/*M* (*SD*)
Sample size (*n*)	546	100.00
Gender		
Women	477	87.36
Men	69	12.64
Place of residence		
City	396	72.53
Rural areas	150	27.47
Type of residence		
Apartment/family house	389	71.25
Rented flat	141	25.82
Student residence (dormitory)	12	2.20
Room provided by a family member	4	0.7
Family structure of origin		
By both parents	442	80.95
By mother	88	16.12
By father	8	1.47
By grandparents	6	1.10
Foster child	2	0.37
Education		
Primary	3	0.55
Vocational	4	0.73
Secondary (including vocational, upper secondary and post-secondary)	265	48.53
Higher education (bachelor's degree)	147	26.92
Higher master's degree	120	21.98
Doctoral	7	1.28
Marital status		
Married	214	39.19
In a civil partnership	172	31.50
Single	160	29.30
Financial situation (1–5), M (SD)	546	3.64 (0.83)
Housing conditions (1–5), M (SD)	546	4.21 (0.80)
Domestic violence (0–5), M (SD)	546	0.73 (1.19)

Values are presented as frequencies and percentages (rounded to two decimal places) or as means (*M*) and standard deviations (*SD*). Percentages may not sum to 100% due to rounding. Material situation and housing conditions were rated on a 5-point scale (1–5), with higher scores indicating better conditions, whereas the frequency of family violence was assessed on a 0–5 scale, with higher scores indicating greater frequency.

### Measures

Psychosocial competencies were assessed using a proprietary Life Skills Self-Assessment Sheet. The tool was developed based on a review of the literature on psychosocial competencies and life skills (24–26). In the first stage, a pool of items describing four domains of functioning was formulated: interpersonal skills, self-awareness, decision-making, and coping with stress. The final version of the questionnaire included 20 items, divided into four subscales: interpersonal skills (7 items), self-awareness (3 items), decision-making (5 items), and coping with stress (5 items). Responses were given on a five-point Likert scale (1 – strongly disagree, 5 – strongly agree). The results for each domain were calculated as the average of the corresponding items. Sample items included statements such as: “I can clearly communicate my needs to others” (interpersonal skills), “I understand my emotional reactions in difficult situations” (self-awareness), “I analyse the possible consequences before making important decisions” (decision-making) and “I can effectively reduce tension in stressful situations” (coping with stress). The internal reliability of the scales was assessed using Cronbach's α coefficient. High internal consistency was obtained for all subscales: interpersonal skills: α = 0.88, self-awareness: α = 0.81, decision-making: α = 0.84, coping with stress: α = 0.86. Exploratory factor analysis suggested a four-factor structure consistent with the theoretical assumptions of the tool. Because the instrument was developed for the purposes of the present study, its psychometric properties should be interpreted as preliminary and require further validation in independent samples.

Reasons for living were measured using the Reasons for Living Scale (RFL-48) in its Polish adaptation, based on a previously validated cultural adaptation for the Polish population (27). The scale assesses cognitive and emotional factors that protect against suicidal behavior and comprises six subscales: Survival and Coping Beliefs (SCB), Responsibility to Family (RF), Child-Related Concerns (CRC), Moral Objections (MO), Fear of Social Disapproval (FSD) and Fear of Suicide (FS), as well as an overall score. Responses were given on a 6-point Likert scale (1 = “completely unimportant,” 6 = “very important”). Higher scores indicate stronger reasons for living. In the present study, the scale showed high reliability (Cronbach's α = 0.92). The original demographic questionnaire was designed to capture as broadly as possible the social and family determinants of mental health in young adults that are relevant from the perspective of suicide prevention. It included variables related to demographic structure (gender, age, place of origin, level of education, type of education, relationship status, number of children and siblings), housing and economic situation (current form of residence, subjective assessment of housing conditions and financial situation), family context (upbringing structure, presence of suicides and alcohol addiction in the family), as well as selected responses to stress and perceptions of factors potentially triggering a suicidal crisis. The variables used reflect the key components of social determinants of mental health described in the literature, including material resources, living conditions, social support, and stressful experiences in the family environment that may modify risk and protective resources in the area of suicidal behavior. Although the variables included in the present study reflect selected dimensions of social and family context, broader structural determinants such as access to services, institutional support, and wider socio-economic inequalities were beyond the scope of the present survey design.

### Procedure and ethics

The study was conducted in Poland between 1 March and 30 June 2025 using an online questionnaire prepared in Google Forms. Participants were recruited through universities, social media, and thematic online groups targeting young adults. The recruitment procedure corresponded to convenience sampling, as participation was voluntary and based on open invitations distributed across multiple online channels in order to reduce excessive concentration of responses within a single social subgroup. It was not possible to precisely determine the number of people who received the invitation to participate in the study due to the open nature of recruitment in the online environment. The study included people who met the following criteria: age between 18 and 35 and informed consent to participate in an anonymous online survey. The exclusion criteria were: refusal to participate in the study, underage or incomplete questionnaire. Before starting the study, respondents received detailed information about its purpose, anonymity rules and data processing methods; participation was voluntary, with the option to discontinue the survey at any time without giving a reason. Data gaps were random and did not exceed a small percentage of observations. Statistical analyses were performed only on cases with a complete set of data for the variables included in a given model. The research procedure was approved by the local Bioethics Committee (No. KB-006.028.2025 of 21 February 2025). The study was conducted in accordance with the principles of the Declaration of Helsinki and the applicable ethical standards for research involving human subjects. Although the study did not involve direct measurement of suicidal thoughts, intentions or behaviors, its subject matter could be emotionally charged, so particular emphasis was placed on voluntary participation, anonymity and the possibility of withdrawing from the study at any stage. Participants were also informed that, if needed, they could contact publicly available psychological or mental health support services in the event of emotional discomfort related to participation.

### Statistical analysis

Statistical analyses were performed using Statistica software (TIBCO Software Inc., Palo Alto, CA, USA). A level *of p* < 0.05 (two-tailed tests) was adopted as the criterion for statistical significance. Descriptive statistics were calculated for all variables and distributions were assessed using the Shapiro–Wilk test. Categorical variables were recoded as dummy variables, while education was treated as an ordinal variable. Spearman's rank correlation coefficients were used for a preliminary assessment of the relationship between psychosocial competence domains and reasons for living (RFL-48 total score and six subscales). The main method of hypothesis verification was hierarchical multiple linear regression analysis. It was performed separately for each of the seven RFL-48 measures as the dependent variable (overall score and subscales: SCB, FSD, FS, MO, RF, CRC). The first block of each model included control variables: gender, place of origin, level of education, housing status, housing conditions, assessment of financial situation, experience of domestic violence, suicide in the family, and alcohol dependence in the family. The second block introduced four domains of psychosocial competence: interpersonal skills, self-awareness, decision-making, and coping with stress. The stability of the models was assessed on the basis of the change in the coefficient of determination (Δ*R*^2^) and its statistical significance. For each regression model, residual plots were additionally inspected to verify approximate homoscedasticity and linearity between predictors and dependent variables. No substantial deviations indicating serious heteroscedasticity were observed. Because the data were cross-sectional and each participant contributed one independent observation, autocorrelation of residuals was not considered a major threat to model validity. Changes in explained variance (ΔR^2^) were interpreted as effect-size indicators of the additional contribution of psychosocial competence domains in successive blocks of the models. Education was retained as an ordinal predictor because the categories reflected a natural progression of educational attainment and preliminary analyses did not indicate non-linear effects requiring separate dummy coding. The impact of individual predictors in the final models was interpreted on the basis of standardized regression coefficients (β). Collinearity was assessed using variance inflation factors (VIF); for all predictors, VIF values remained below 3 (max. VIF = 2.7), confirming the absence of a strong collinearity problem. Despite deviations from normal distribution for some variables, the distributions were considered acceptable based on the analysis of Q–Q plots and skewness and kurtosis values. Due to the large sample size (N = 546), the linear regression estimation was assumed to be robust to moderate violations of normality assumptions.

## Results

The socio-demographic characteristics of the sample are presented in Table 1. The results of the analyses of the relationships between psychosocial competence domains and reasons for living, taking into account the role of social and family factors, are presented below.

### Relationships between psychosocial competencies and reasons for living, taking into account social factors

In order to verify the hypotheses, seven hierarchical linear regression analyses were performed, in which the dependent variables were the overall RFL-48 score and its six subscales (SCB, FSD, FS, MO, RF, CRC). In all models, the first block included socio-demographic and family control variables, while the second block introduced four domains of psychosocial competencies: interpersonal skills, self-awareness, decision-making, and coping with stress. The addition of competence domains in all seven models resulted in a statistically significant increase in explained variance (Δ*R*^2^ from 0.023 to 0.295; all *p* ≤ 0.013; Table 2), indicating that psychosocial competence domains remain significantly associated with reasons for living even after controlling for social and family factors. The final model for the overall RFL-48 score explained 27% of the variance (adjusted *R*^2^ = 0.252). The highest adjusted R^2^ value was obtained for the SCB subscale (0.334), while for FSD (0.026) and FS (0.046) the model fit was low, although statistically significant (Table 2).

**Table 2:** Fit of hierarchical regression models explaining the overall level of reasons for living (RFL-48) and its subscales after controlling for sociodemographic and family factors.

Dependent variable	*N*	Final model *R^2^*	Adjusted *R^2^*	*ΔR^2^* (competence block)	*F* changes (df1, df2)	*p*
RFL-48 sum	546	0.270	0.252	0.221	40.21 (4, 532)	< 0.001
SCB	546	0.350	0.334	0.295	60.49 (4, 532)	< 0.001
FSD	546	0.049	0.026	0.032	4.51 (4, 532)	0.0014
FS	546	0.068	0.046	0.035	5.03 (4, 532)	< 0.001
MO	546	0.098	0.076	0.051	7.57 (4, 532)	< 0.001
RF	546	0.153	0.133	0.115	18.13 (4, 532)	< 0.001
CRC	546	0.068	0.045	0.023	3.23 (4, 532)	0.0122

Δ*R*^2^ and *F* change refer to the increase in explained variance after adding the four psychosocial competence domains. SCB, Survival and Coping Beliefs; RF Responsibility to Family; CRC, Child-Related Concerns; MO, Moral Objections; FSD, Fear of Social Disapproval; FS, Fear of Suicide.

### Psychosocial domains of competence as the strongest predictors of reasons for living

In the final models, psychosocial competence domains showed the strongest associations with reasons for living compared to the socio-demographic factors included (Table 3). For the overall RFL-48 score, self-awareness was most strongly associated (β = 0.284; *p* < 0.001), followed by interpersonal competencies (β = 0.178; *p* < 0.001) and decision-making (β = 0.109; *p* = 0.039). With regard to SCB, the strongest predictor was self-awareness (β = 0.294; *p* < 0.001), with a significant but weaker association with coping with stress (β = 0.108; *p* = 0.0453). For FS, the strongest relationship was observed for coping with stress (β = −0.227; *p* < 0.001). With regard to hypothesis H2, the RF subscale was significantly predicted by self-awareness (β = 0.150; *p* = 0.0177) and decision-making (β = 0.203; *p* < 0.001). For FSD, significant correlations were found for self-awareness (β = 0.166; *p* = 0.0128) and interpersonal competence (β = 0.153; *p* = 0.0112). For the MO subscale, self-awareness showed the strongest correlation (β = 0.212; *p* = 0.0011). In the case of CRC, none of the analyzed competency predictors achieved statistical significance in the final model.

**Table 3:** Final models explaining reasons for living: psychosocial competence domains and sociodemographic and family factors as predictors (standardized β; *p*).

Predictor	RFL-48 sum	SCB	FSD	FS	MO	RF	CRC
Gender (male)	−0.029 (0.4560)	0.010 (0.7890)	0.030 (0.4951)	−0.123 (0.0050)	−0.019 (0.6622)	−0.031 (0.4574)	−0.011 (0.8075)
Place of origin (rural)	0.030 (0.4268)	0.019 (0.6039)	−0.032 (0.4651)	−0.001 (0.9747)	0.081 (0.0588)	0.048 (0.2440)	0.007 (0.8741)
Education (level)	0.001 (0.9864)	−0.027 (0.4624)	−0.018 (0.6862)	0.043 (0.3352)	0.017 (0.7010)	0.012 (0.7789)	0.038 (0.3880)
Marital status (in a relationship)	0.064 (0.0975)	0.047 (0.1932)	0.017 (0.6986)	−0.031 (0.4725)	0.057 (0.1804)	0.084 (0.0421)	0.125 (0.0041)
Form of residence (outside family home)	−0.020 (0.6146)	0.047 (0.2059)	0.005 (0.9186)	−0.072 (0.1040)	−0.098 (0.0249)	−0.039 (0.3613)	−0.083 (0.0624)
Financial situation (1–5)	0.001 (0.9844)	0.015 (0.7076)	−0.057 (0.2382)	−0.015 (0.7505)	−0.023 (0.6227)	−0.017 (0.7112)	0.068 (0.1544)
Housing conditions (1–5)	0.014 (0.7358)	0.011 (0.7908)	0.052 (0.2838)	0.074 (0.1257)	−0.019 (0.6848)	−0.013 (0.7814)	−0.073 (0.1323)
Domestic violence (0–5)	−0.061 (0.1196)	−0.057 (0.1179)	−0.071 (0.1122)	−0.022 (0.6178)	−0.031 (0.4702)	−0.071 (0.0896)	0.047 (0.2898)
Alcohol in the family (yes)	0.096 (0.0129)	0.128 (< 0.001)	0.006 (0.8879)	−0.021 (0.6354)	−0.045 (0.2948)	0.107 (0.0104)	0.067 (0.1226)
Interpersonal skills	0.178 (< 0.001)	0.154 (0.0020)	0.153 (0.0112)	0.117 (0.0501)	0.099 (0.0915)	0.091 (0.1106)	0.081 (0.1762)
Self-awareness	0.284 (< 0.001)	0.294 (< 0.001)	0.166 (0.0128)	0.061 (0.3528)	0.212 (0.0011)	0.150 (0.0177)	0.110 (0.0955)
Decision-making	0.109 (0.0393)	0.117 (0.0198)	−0.100 (0.0974)	0.107 (0.0731)	−0.109 (0.0650)	0.203 (< 0.001)	0.007 (0.9108)
Coping with stress	−0.003 (0.9528)	0.108 (0.0453)	−0.083 (0.2009)	−0.227 (< 0.001)	0.020 (0.7574)	−0.045 (0.4631)	−0.026 (0.6832)

β denotes standardized regression coefficients from the final model (controls and competence variables). Values in parentheses are *p* values. SCB, Survival and Coping Beliefs; RF, Responsibility to Family; CRC, Child-Related Concerns; MO, Moral Objections; FSD, Fear of Social Disapproval; FS, Fear of Suicide.

### The specificity of the impact of socio-demographic factors on individual dimensions of reasons for living

The analysis of the final models showed that the impact of socio-demographic factors on reasons for living was selective (Table 3). Gender was a significant predictor only for the FS dimension, with men scoring lower than women (β = −0.123; *p* = 0.005). Marital status was positively associated with RF (β = 0.084; *p* = 0.042) and CRC (β = 0.125; *p* = 0.004). Living arrangements outside the family home were significantly associated with MO (β = −0.098; *p* = 0.025). The presence of alcohol dependence in the family was positively associated with the overall RFL-48 score (β = 0.096; *p* = 0.013), SCB (β = 0.128; *p* < 0.001), and RF (β = 0.107; *p* = 0.010). The remaining variables did not show significant associations with reasons for living in the final models.

## Discussion

The results indicate that psychosocial competencies may represent an important psychosocial resource associated with reasons for living in young adults. Although the observed associations were consistent across several models, they should be interpreted with caution due to the cross-sectional design of the study. The present findings do not demonstrate causal relationships between psychosocial competencies and reasons for living; rather, they indicate that these constructs co-occur in meaningful ways within the analyzed socio-demographic and family context. At the same time, the observed relationships persisted after controlling for socio-demographic factors, suggesting that an individual's psychosocial resources remain an important component of processes conducive to mental health. These results are in line with the current trend in research on mental resilience and protective resources as key elements in the prevention of suicidal behavior (10, 12, 22).

### The role of psychosocial competencies in shaping reasons for living

The results of the analyses indicate that individual domains of psychosocial competence are differentially associated with specific dimensions of reasons for living. The particularly clear relationship between self-awareness and the overall level of reasons for living suggests that the ability to reflectively understand one's own emotional states and needs may co-occur with more stable cognitive and emotional resources that support the maintenance of motivation to live. This result is consistent with the literature indicating that developed self-awareness facilitates adaptive processing of stressful experiences and promotes more flexible coping strategies (12, 28). One possible interpretation is that self-awareness may be associated with regulatory processes facilitating recognition of internal emotional states, reflective appraisal of stress, and more adaptive selection of coping responses; however, these mechanisms were not directly tested in the present study (29). In the context of hypothesis H1, the results obtained indicate its partial confirmation: as assumed, the domains of decision-making and coping with stress were significantly related to the level of reasons for living, but self-awareness proved to be the strongest predictor of the overall level of reasons for living. At the same time, stress management skills showed a particularly strong relationship with survival and coping beliefs (SCB) and fear of suicide (FS), while self-awareness and interpersonal skills were important in the case of FSD. This may indicate that cognitive-decision-making and regulatory abilities may be an important component of protective resources, promoting the interpretation of difficult situations as solvable and associated with a higher sense of agency. This interpretation is consistent with transdiagnostic models of suicidal behavior, which emphasize the role of cognitive and emotional processes in shaping risk and resilience (8, 9, 22). This interpretation may also be viewed as theoretically consistent with the Interpersonal Theory of Suicide; however, variables central to that model, such as perceived burdensomeness and thwarted belongingness, were not directly measured in the present study (21).

### Psychosocial protective mechanisms against suicidal behavior

According to hypothesis H2, self-awareness and decision-making skills played a particularly important role in relation to the dimension of family responsibility (RF), while in the case of moral doubts (MO), self-awareness remained the only significant predictor. These results emphasize the importance of social relationships, moral norms and a sense of responsibility for others as factors that can act as barriers to suicidal behavior. This is consistent with the socio-cultural perspective on suicidal behavior, which emphasizes the importance of social ties and family commitments as key protective factors (30–32). The particular importance of coping with stress for survival beliefs and coping ability (SCB) is consistent with earlier findings indicating that effective emotion regulation and the ability to manage psychological stress may be associated with lower levels of suicidal ideation even in the presence of other risk factors (33, 34).

### Socio-demographic determinants of reasons for living

The obtained relationships indicate that a higher level of psychosocial competence co-occurs with a higher level of reasons for living even after taking into account socio-demographic factors, which emphasizes the importance of these resources in the structure of factors related to the mental wellbeing of young adults. At the same time, the influence of social and family factors in the analyzed models proved to be selective and limited to selected dimensions of reasons for living, which suggests that individual resources may play a relatively stronger role in shaping these relationships. This may partly reflect the fact that psychosocial competencies represent more proximal and modifiable individual resources directly involved in everyday cognitive and emotional regulation, whereas socio-demographic variables often operate indirectly through broader contextual mechanisms. This interpretation is consistent with the approach to social determinants of mental health, which emphasizes the complex and multi-level nature of factors influencing wellbeing (11, 12). The observed gender differences, while maintaining the importance of psychosocial competencies, are consistent with earlier findings on gender differences in suicidal behavior (30, 35, 36) and point to the need to consider the gender perspective when designing preventive interventions.

### Implications for public health policy and preventive practice

The results suggest that psychosocial competencies are an important component of the mental functioning of young adults and may be a useful reference point in the design of mental health promotion activities, but these conclusions require further verification in longitudinal and intervention studies. From a practical perspective, the results suggest that the development of psychosocial competencies may be considered as one of the potentially beneficial elements of activities promoting the mental health of young adults, although the effectiveness of such interventions was not directly tested in the present study and requires empirical verification in broader and more diverse populations. Such activities may potentially include structured psychoeducational workshops, university-based mental health programmes, peer-support initiatives, and skills-oriented preventive interventions focused on emotional regulation, decision-making, and interpersonal communication. Early strengthening of these resources may act as a potential buffer against developmental and social stressors characteristic of this stage of life, although this assumption requires confirmation in intervention studies (6, 37, 38).

### Limitations and directions for further research

This study has several important limitations that should be taken into account when interpreting the results. First, its cross-sectional nature does not allow for conclusions to be drawn about causal mechanisms or for an unambiguous assessment of the direction of the relationship between psychosocial competencies and reasons for living. Therefore, the regression coefficients observed in the present models should be interpreted as indicators of contemporaneous statistical associations rather than predictors of longitudinal change or developmental effects over time. Furthermore, the recruitment of participants using online tools may have led to an overrepresentation of people with higher levels of education and urban residents. In addition, self-selection bias cannot be excluded, as individuals more interested in psychosocial functioning or mental health topics may have been more willing to participate in the study. This sampling pattern may have favored participation of individuals with relatively higher psychosocial competencies, particularly among respondents with higher education and urban residence, which should be considered when interpreting the observed associations. Combined with a clear predominance of women in the sample, this limits the representativeness of the results in relation to the young adult population and makes it difficult to generalize them. This gender imbalance requires caution when interpreting sex-related differences, as psychosocial competencies and reasons for living may partly vary across gender groups. An additional limitation is the use of self-reported data, which may be subject to distortions resulting from the tendency toward social approval and subjective interpretation of one's own experiences. In addition, the present analyses were based on linear regression models and did not examine potential non-linear relationships or moderation effects by socio-demographic variables, which should be explored in future studies. The study was deliberately focused on the young adult population, which is reflected in the title of the article and the design of the research tools used. This means that the results obtained refer to the specifics of this phase of life and cannot be directly transferred to other stages of development. Furthermore, the adopted research design does not allow for an analysis of the dynamics of changes in reasons for living and psychosocial competencies over time, which limits the possibility of more fully capturing their long-term significance for suicidal behavior. It should also be noted that protective factors may take different forms depending on the cultural, religious and social context, which was not the subject of in-depth analysis in this study. Future studies should use longitudinal designs, a multi-method approach and more representative samples, as well as conduct analyses in diverse cultural and social populations. It would also be valuable to evaluate intervention studies aimed at strengthening psychosocial competencies in order to determine whether their development contributes empirically to stronger protective factors related to reasons for living. It should also be emphasized that the study focused on cognitive and emotional reasons for living as protective factors, rather than actual suicidal behavior or suicidal thoughts, which limits the possibility of drawing direct conclusions about the prevention of suicide attempts.

## Conclusions

The results obtained indicate that psychosocial competencies remain significantly related to the level of reasons for living in young adults, and the observed relationships persist even after taking into account selected socio-demographic and family conditions. However, due to the cross-sectional design, these associations should not be interpreted as evidence of causal influence, but rather as statistically co-occurring relationships requiring confirmation in longitudinal studies. This suggests that psychosocial competencies may constitute an important element of protective resources functioning in a specific socio-demographic and family context, meaning that individual competencies remain relevant even when social and family conditions are less supportive, although these contextual factors still contribute to the broader pattern of mental health resources. The findings underscore the relevance of further research on the mechanisms of strengthening the mental health of young adults from the perspective of social determinants of health, as well as on the design and evaluation of the effectiveness of interventions aimed at developing these resources. These findings may also support the development of mental health promotion programmes focused on psychosocial competencies, implemented in educational institutions, community settings, and preventive public health practice.

## Data Availability

The raw data supporting the conclusions of this article will be made available by the authors, without undue reservation.
